# Fairness in household financial contribution to the Iran’s healthcare system from 2008 to 2018

**DOI:** 10.1186/s13104-021-05606-8

**Published:** 2021-05-17

**Authors:** Ali Darvishi, Mostafa Amini-Rarani, Mohammad Hossein Mehrolhassani, Vahid Yazdi-Feyzabadi

**Affiliations:** 1 Students’ Scientific Research Center (SSRC), Tehran University of Medical Sciences (TUMS), Tehran, Iran; 2 Department of Health Management and Economics, School of Public Health, Tehran University of Medical Sciences, Tehran, Iran; 3 Social Determinants of Health Research Center, Isfahan University of Medical Sciences, Isfahan, Iran; 4 Social Determinants of Health Research Center, Institute for Futures Studies in Health, Kerman University of Medical Sciences, Kerman, Iran; 5 Health Services Management Research Center, Institute for Futures Studies in Health, Kerman University of Medical Sciences , 7616913555 Kerman, Iran; 6 Department of Health Management, Policy and Economics, Faculty of Management and Medical Information Sciences , Kerman, Iran

**Keywords:** Fair Financial Contribution Index (FFCI), Financial protection, Iran’s health transformation program (HTP)

## Abstract

**Objective:**

Enhancing financial protection in health is one of the main goals of Iran’s health transformation program (HTP), a recent reform conducted in early 2014. This study aimed to measure financial protection using the fair financial contribution index (FFCI) in urban and rural areas before (2008–2013) and after (2014–2018) the HTP implementation. Using a retrospective study on annual national cross-sectional surveys of households' income and expenditure, FFCI was measured. The total sample sizes for urban and rural areas from 2008 to 2018 were 207,980 and 212,249 households, respectively.

**Results:**

The worst fair contributions to health expenditure in urban (FFCI = 0.684) and rural areas (FFCI = 0.530) were related to 2010 and 2009, respectively. Otherwise, the best fair contributions for urban (FFCI = 0.858) and rural (FFCI = 0.836) areas were made in 2011. Before the HTP implementation began, FFCI showed minor changes from 0.834 in 2008 to 0.833 in 2013. Following the HTP implementation, the FFCI values in urban and rural populations declined (worsened) from 0.842 to 0.836 and 0.816 to 0.809, respectively.On average more fair financial contributions had been made following five years after the HTP, especially in rural areas, but less than that expected in upstream documents (as determined 0.9).

## Introduction

Equity in utilization and resource distribution is an essential intermediate objective of health financing policy [1]. The achievement of this objective leads to health gains and financial risk protection, especially for those in need. Since 2000, the WHO has drawn attention to ensure equitable (or fair) financing for health care, highlighting that health systems are not just about improving health status. However, also improving fairness through health system financing and delivery has a broader social value [2]. In the 2010 World Health Report, the fair financial contribution was considered to be the main criterion for achieving universal health coverage. [3].

Generally, different methods have been used to finance health systems, including tax-based insurance, social insurance, private insurance, and Out-of-Pocket (OOP) payment [4]. From the viewpoint of both risk protection and equity, OOP payments is considered to be the worst possible form of health financing [5, 6]. Evidence shows that the high share of OOP payments for health services is a sign of inadequate social health coverage and can lead to economic hardship, particularly in low-and middle-income countries [7, 8]. OOP imposes the most significant burden on the poor and carries a high risk of falling households into poverty by imposing Catastrophic Health Expenditure (CHE) [9]. To this end, WHO has more than ever emphasized the CHE and the Fair Financial Contribution Index (FFCI) as equity indicators for household financial contributions to health systems [10, 11] and recommended the universal health coverage strategy with the aim of ensuring that all people have access to the required health services without any financial difficulties at the time of receiving services. Fairness in healthcare financing is assessed by the level of inequality in the payment of health care between households of unequal Capacity to Pay (CtP) [12].

Overall, equitable financing is a crucial objective of healthcare systems and healthcare financial risk protection, recommended to be measured on a continuous and periodic basis every 2–5 years [5, 13].

In Iran, given the requirements of the National Five-Year Development Plans to address high OOP rates, i.e., 52.1% of total health expenditure [14], the increased contribution of the government in providing the funds for public health expenses and appropriate allocation of public resources in the health sector are of great importance. Accordingly, in May 2014, the Ministry of Health and Medical Education (MoHME) of Iran implemented a comprehensive health system transformation package called the Health Transformation Plan (HTP) [15]. The reduction in the percentage of OOP payments and the reduction in the percentage of households facing CHE were identified as top priorities in HTP content.

The present study aims to how the HTP achieved its objectives in terms of fair financial protection by assessing FFCI in various households of urban and rural areas before (2008–2013) and after (2014–2018) the implementation of the HTP.

## Main text

### Methods

The present study is a retrospective descriptive study that was conducted using annual national repeated cross-sectional surveys on households' income and expenditure from 2008 to 2018 in Iran.

Iran's household expenditure and income survey plan have been implemented in rural areas since 1963 and urban areas since 1968 by Iran Statistical Center (ISC). In addition to household expenditure, income information has also been collected since 1974 and has been given annually to date. In this plan, information related to the average expenditures and income of an urban and rural household in the country is provided annually. Data obtained from this annual survey are collected using a questionnaire for about 38,000 Iranian households that have been sampled and entered into the survey in a step-by-step manner. The questionnaire includes three sections, including socio-economic characteristics of the household, expenses (household food and non-food expenses), and household income. The pillars studied in this survey are the educational status of the household, household assets, access to basic facilities and public goods and household expenses in various sectors such as Health [16].

The sample size of the data used in the study is presented in the form of Table 1 separately for each year and the urban and rural population.

**Table 1:** Sample size in study years

Year	Urban	Rural	Total
2018	19,382	19,708	39,090
2017	18,666	18,204	36,870
2016	18,702	19,585	38,287
2015	18,728	19,787	38,515
2014	18,536	19,658	38,194
2013	18,881	19,437	38,318
2012	18,886	19,391	38,227
2011	18,872	19,382	38,254
2010	18,809	19,340	38,149
2009	18,205	19,207	37,412
2008	20,313	18,550	38,863
Total	207,980	212,249	420,179

In order to calculate and compare households' fair financial contribution in health expenditures, the distribution of household financial contributions among them is summarized using an index called the FFCI. This index puts much weight on households that spend a high proportion of their income on health. FFCI generally reflects inequality in the financial contribution of households in health, although it reflects explicitly households that face catastrophic health expenditures. The mathematical cube-root is used to place more weight on households in the distribution sequence. In this study, the following equation has been used to calculate the values of health expenditures FFCI.

$${\varvec{FFCI}}=1-\sqrt[3]{\frac{\sum_{{\varvec{h}}=1}^{{\varvec{n}}} {{\varvec{w}}}_{{\varvec{h}}} \left/ {{\varvec{oopctp}}}_{{\varvec{h}}} \right.-{{\varvec{oopctp}}}_{0}/3}{\sum {{\varvec{w}}}_{{\varvec{h}}}}}$$

where *w*_h_ represents households sampling weights, *oopctp*_*h*_ represents the ratio of OOPs to the household's CtP for health expenditures, which shows the financial burden borne for health services. The steps and details of the calculation of CtP are presented in another study by the researchers [17, 18]. Also, the value of *oop*_*0*_ in the above Formula is obtained from the following equation:

$${{\varvec{o}}{\varvec{o}}{\varvec{p}}}_{0}=\frac{\sum {{\varvec{w}}}_{{\varvec{h}}}{\times {\varvec{o}}{\varvec{o}}{\varvec{p}}}_{{\varvec{h}}}}{\sum {{\varvec{w}}\times {\varvec{c}}{\varvec{t}}{\varvec{p}}}_{{\varvec{h}}}}$$

FFCI calculations were performed for six years before the HTP implementation and five years after it, and its trend in these years was reviewed and analyzed. Excel 2016 and Stata 14 software were used to prepare the data and analyze the results.

### Results

The total sample sizes for urban and rural areas from 2008 to 2018 are shown in Table 1. According to the sample analyzed, 207,980 and 212,249 of households lived in urban and rural areas, respectively.

The FFCI results before and after the HTP in rural, urban, and the total population are shown in Table 2. The worst fair contributions to health expenditure in urban (FFCI = 0.684) and rural areas (FFCI = 0.530) were occurred in 2010 and 2009, respectively. Otherwise, the best fair contributions for urban areas (FFCI = 0.858) and rural areas (FFCI = 0.836) were made in 2011. During 2008 and 2009, the values of FFCI for urban, rural and total population were reduced and worsened from 0.842 to 0,750, 0.821 to 0.530, and 0.834 to 0.657, respectively. In comparison, the FFCI values were increased in 2010, except for urban areas. Before the HTP implementation (between 2008 and 2013), FFCI witnessed minor changes from 0.834 in 2008 to 0.833 in 2013. Following the HTP implementation (between 2014 and 2018), the FFCI values in urban, rural, and the total population declined (worsened) from 0.842 to 0.836, 0.816 to 0.809, and 0.833 to 0.829, respectively. Consequently, during the five years of HTP implementation from 2014 to 2018, the FFCs have slightly worsened by 0.71%, 0.85%, and 0.48% in urban, rural, and total population, respectively.

**Table 2:** FFCI Values in Urban and Rural Iranian Households and Mean Value before and after the HTP

	Year	Urban	Rural	Total
Before the HTP	2008	0.842	0.821	0.834
	2009	0.75	0.53	0.657
	2010	0.684	0.78	0.703
	2011	0.858	0.836	0.85
	2012	0.855	0.828	0.846
	2013	0.841	0.817	0.833
	Mean	0.805	0.769	0.787
After the HTP	2014	0.842	0.816	0.833
	2015	0.842	0.822	0.835
	2016	0.839	0.823	0.833
	2017	0.84	0.821	0.834
	2018	0.836	0.809	0.829
	Mean	0.84	0.818	0.833
Mean 11 years		0.82	0.791	0.807

**Fig. 1:**
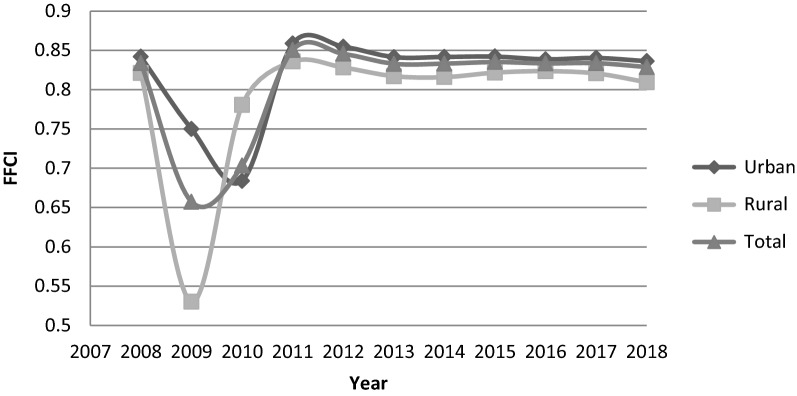
Fair financial contribution to health expenditure in Iran (2008–2018)

Figure 1 depicts changes in FFCIs of Iranian households to health expenditure for the years 2008 to 2018. Throughout this period, the best and the worst indices are belonging to urban areas in 2011and rural areas in 2009, respectively. The rise of the FFCI value in the first year of HTP implementation in 2014 almost stopped the declining trend that started in 2013. After a slight rise in 2015, the overall FFCI stayed almost constant in 2015–2017 and then decreased in 2018. The pattern of improvement of the FFCI for residence in rural areas occurred dramatically after the introduction of the HTP up to 2016, but undesirably declining in the last two years. In contrast, the FFCIs for urban areas underwent small improvements following the HTP.

### Discussion

The FFCI can help policymakers recognize the flaws in the financial protection mechanisms embedded in the health financing system. According to our 11-year analysis, it is clear that financial contributions in Iranian households on average were 21% (Mean FFCI = 0.79) and 18% (Mean FFCI = 0.82) away from achieving complete fairness in rural and urban areas, respectively. Also, the results showed that despite the decline in FFCI in 2018, on average more fair financial contributions had been made following five years after the HTP, particularly in rural areas compared with average six years before HTP. In addition, FFCIs have shown higher inequalities in rural areas compared to urban areas both before and after the HTP.

Previous studies from different regions of Iran have been reported FFCI values ranging from 0.83 in a national study [19] to 0.6 in a study conducted in Shiraz province in 2012 [4] or 0.56 in Kermanshah province in 2005 [20]. In 2012, Raghfar et al. reported FFCI levels with slight fluctuations in 1984–2010. The FFCI values were 0.76 and 0.79 in the rural and urban sectors in 1984, respectively. In 2010, it was 0.79 in both the rural and urban sectors [21]. In their study, Fazaeli et al. showed that the FFCI decreased from 0.841 in 2003 to 0.827 in 2010, and this trend showed a decline in the fair financial contribution to health expenditure. [22]. The results of the study conducted by Mousavi et al. also showed that FFCI of Iranian households ranged from 0.83 in 2009 to 0.91 in 2014 and experienced minor fluctuations from 1989–2014. In this study, the lowest FFCI value was measured in 2009, in line with the current study [23].

Results on the trend of FFCI after HTP compared to before its implementation has shown that on average, improvements in FFCI values have occurred. However, by examining this index from year to year, it is clear that the numerical values of the index did not change significantly in 2018 compared to 2013 and were almost constant. So, consistent with the Kheibari et al.' study [19] could be stated that fair contribution to financing improved after the HTP but less than that expected. The reasons behind the small improvements in the FFCI could be explained as follows. First, the HTP focuses only on inpatient services delivered in hospitals affiliated with medical universities under the sponsorships of the MoHME. Accordingly, outpatient and inpatient services provided in other hospitals and the private sector, which have a high proportion of services almost not considered being part of the HTP. Second, despite the existence of clinical guidelines for various healthcare services, but in practice, healthcare services are not provided based on these guidelines, which may increase the cost of treatment and increase OOP payments. Third, as part of the HTP, the relative value of health care services has been increased to motivate healthcare providers to deliver high-quality services [24] according to the new relative value book [25]. Accordingly, new medical tariffs ascended the absolute amounts of OOP payments irrespective of reduction in payment for inpatient services. Forth, the demand for healthcare services delivered in the public sectors has been stimulated by the HTP and, due to human, financial, and equipment constraints, many people in the public sector (such as public hospitals) have needlessly been referred to the private sector. This has doubled the financial burden on households.

### Conclusion

We concluded that there is a difference between present level of FFCI and the targeted amounts (as determined 0.9) in Iran’s national development plans. Also, FFCI has not been improved during the years after the implementation of HTP than before ones and this necessitates the strengthen actions and interventions to reduce and achieve to desired level recommended by the WHO for the OOP spending (Up to 15–20%).

## Limitations

This study also had some laminations. First, the FFCI inherent drawback is that it represents both vertical and horizontal equity, without distinguishing between them, though, as Wagstaff (2002) declared [26], the two suggest very different policy implications. Another limitation is that the FFCI cannot distinguish between progressive and regressive health financing. This issue necessitates using of other indices such as the Kakwani index as a decomposable approach. The expenditures data could have a potential risk of reporting bias (overestimation of expenditures due to telescoping or underestimation due to forgetting) [27], which was controlled by shortening the recall period's length. Also, to acquire estimations more representative of the whole year, the samples are evenly distributed among the year's months. This study was also conducted at the national level. Details at the provincial level could enhance the results.

## Data Availability

All data obtained during this study is included in this article. The datasets used and/or analysed during the current study are available from the corresponding author on reasonable request.

## Abbreviations

FFCI: Fair Financial Contribution Index
HTP: Health Transformation Program
OOP: Out-of-Pocket
CHE: Catastrophic Health Expenditure
CtP: Capacity to Pay
